# Supplementary material for: Single cell atlas for 11 non-model mammals, reptiles and birds
Source: Nat Commun. 2021 Dec 6;12:7083. doi: 10.1038/s41467-021-27162-2 (PMC8648889; doi:10.1038/s41467-021-27162-2)
Supplement: Supplementary file 1 — Supplementary Information [file 41467_2021_27162_MOESM1_ESM.pdf]

Single cell atlas for mammals, reptiles and birds

Dongsheng Chen[1,23], Jian Sun[2,3,23], Jiacheng Zhu[1,4,23], Xiangning Ding[1,4,23], Tianming Lan[1,5,23], Xiran Wang[2,3,23], Weiying Wu[1], Zhihua Ou[1], Linnan Zhu[1], Peiwen Ding[1,4], Haoyu Wang[1,4], Lihua Luo[1,4], Rong Xiang[1,4], Xiaoling Wang[1,4], Jiaying Qiu[1,4], Shiyou Wang[1,4], Haimeng Li[1,4], Chaochao Chai[1,4], Langchao Liang[1,4], Fuyu An[6], Le Zhang[7], Lei Han[7], Yixin Zhu[1,4], Feiyue Wang[1], Yuting Yuan[1], Wendi Wu[1], Chengcheng Sun[1,4], Haorong Lu[8,9], Jihong Wu[10,11,12], Xinghuai Sun[10,11,12], Shenghai Zhang[10,11,12], Sunil Kumar Sahu[1], Ping Liu[1], Jun Xia[1], Lijing Zhang[1,4], Haixia Chen[1,4], Dongming Fang[1], Yuying Zeng[1,4], Yiquan Wu[13], Zehua Cui[2,3], Qian He[2,3], Sanjie Jiang[1], Xiaoyan Ma[14], Weimin Feng[1], Yan Xu[1], Fang Li[15], Zhongmin Liu[15], Lei Chen[16], Fang Chen[1], Xin Jin[1], Wei Qiu[17], Tianjiao Wang[18], Yang Li[18], Xiumei Xing[18], Huanming Yang[1,19], Yanchun Xu[7,20],Yan Hua[6], Yahong Liu[2,3], Huan Liu[1,4, 21], Xun Xu[1,22]

1. BGI-Shenzhen, Shenzhen 518083, China
2 . National Risk Assessment Laboratory for Antimicrobial Resistance of Animal Original Bacteria, South China Agricultural University, Guangzhou 510642, China
3 . Guangdong Laboratory for Lingnan Modern Agriculture, Guangzhou 510642, China
4 . College of Life Sciences, University of Chinese Academy of Sciences, Beijing 100049, China
5 . Department of Biology, University of Copenhagen, DK-2100 Copenhagen, Denmark
6 . Guangdong Provincial Key Laboratory of Silviculture, Protection and Utilization, Guangdong Academy of Forestry, Guangzhou 510520, China
7 . College of Wildlife Resources Northeast Forestry University. Harbin 150040, China
8 . China National Genebank, BGI-Shenzhen, Shenzhen 518120, China
9 . Shenzhen Key Laboratory of Environmental Microbial Genomics and Application, BGI-Shenzhen, Shenzhen 518120, China
10 . Eye and ENT Hospital, College of Medicine, Fudan University, Shanghai, China
11 . Shanghai Key Laboratory of Visual Impairment and Restoration, Science and Technology Commission of Shanghai Municipality, Shanghai, China
12 . Key Laboratory of Myopia, Ministry of Health, Shanghai, China
13 . HIV and AIDS Malignancy Branch, Center for Cancer Research, National Cancer Institute, National Institutes of Health, Bethesda, MD 20892-1868, USA
14 . Department of Biochemistry, University of Cambridge, Cambridge CB21QW, UK
15 . Research Center for Translational Medicine, East Hospital, Tongji University School of Medicine, 150 Jimo Road, Shanghai 200120, China
16 . College of Veterinary Medicine, Yangzhou University, Yangzhou 225009, China
17 . Department of Neurology, The Third Affiliated Hospital of Sun Yat-Sen University, Guangzhou 510080, China
18 . Institute of Special Animal and Plant Sciences (ISAPS) (ISAPS) of Chinese Academy of Agricultural Sciences, Changchun, China
19 . Guangdong Provincial Academician Workstation of BGI Synthetic Genomics, BGI-Shenzhen, Shenzhen 518120, China
20 . College of Wildlife and Protected Areas, Northeast Forestry University, No. 26, Hexing Road, Xiangfang District, Harbin 150040, China.
21 . State Key Laboratory of Agricultural Genomics, BGI-Shenzhen, Shenzhen 518083, China
22 . Guangdong Provincial Key Laboratory of Genome Read and Write, BGI-Shenzhen 518083, Shenzhen, China
23 . Those authors contributed equally: Dongsheng Chen, Jian Sun, Jiacheng Zhu, Xiangning Ding, Tianming Lan, Xiran Wang

Correspondence should be addressed to X. X. (xuxun@genomics.cn), H. L. (liuhuan@genomics.cn), Y.-H. L. (gale@scau.edu.cn) and Y. H. (wildlife530@hotmail.com).

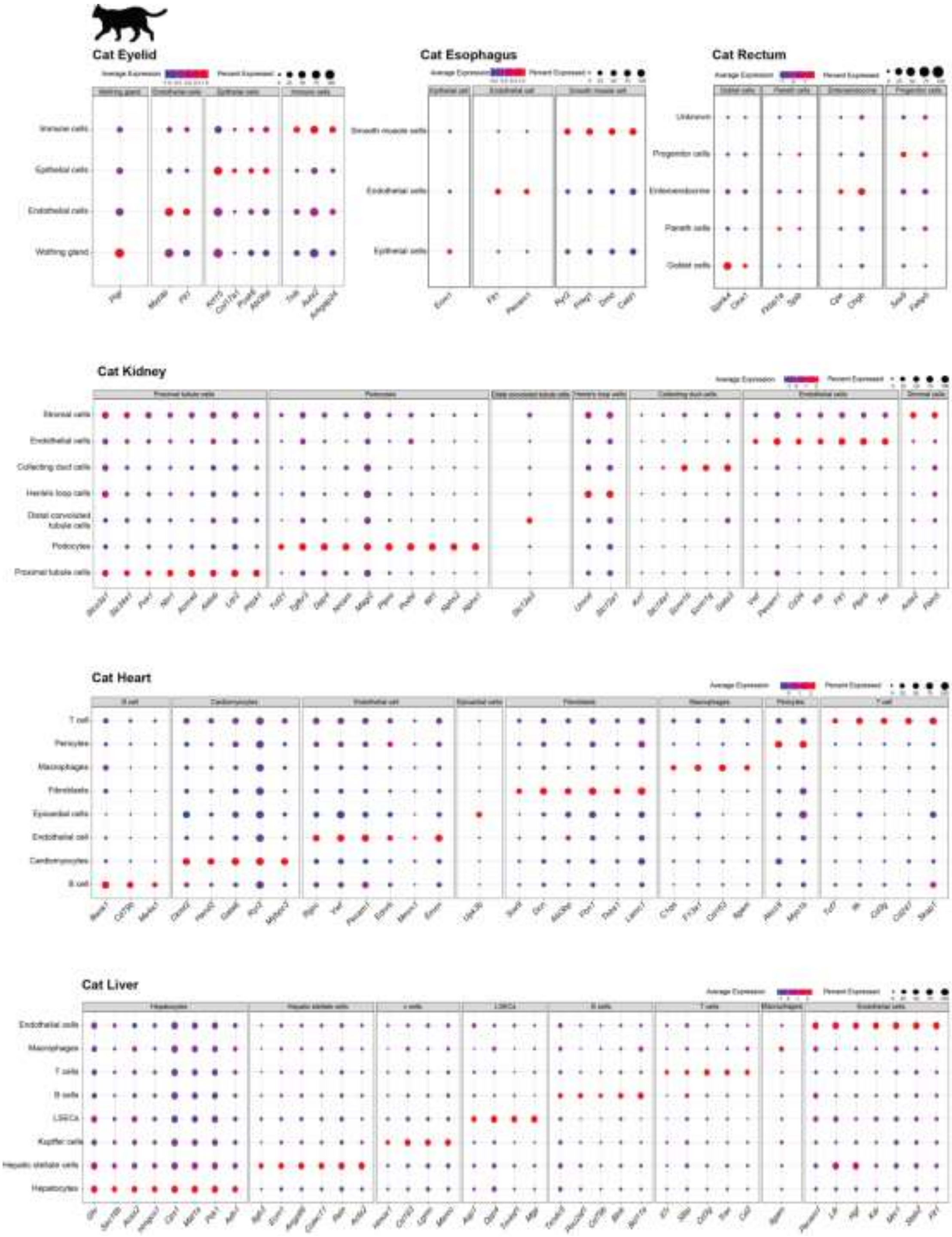

Supplementary Fig. 1. Dot plots showing expression patterns of cell type marker genes in different tissues of cat.

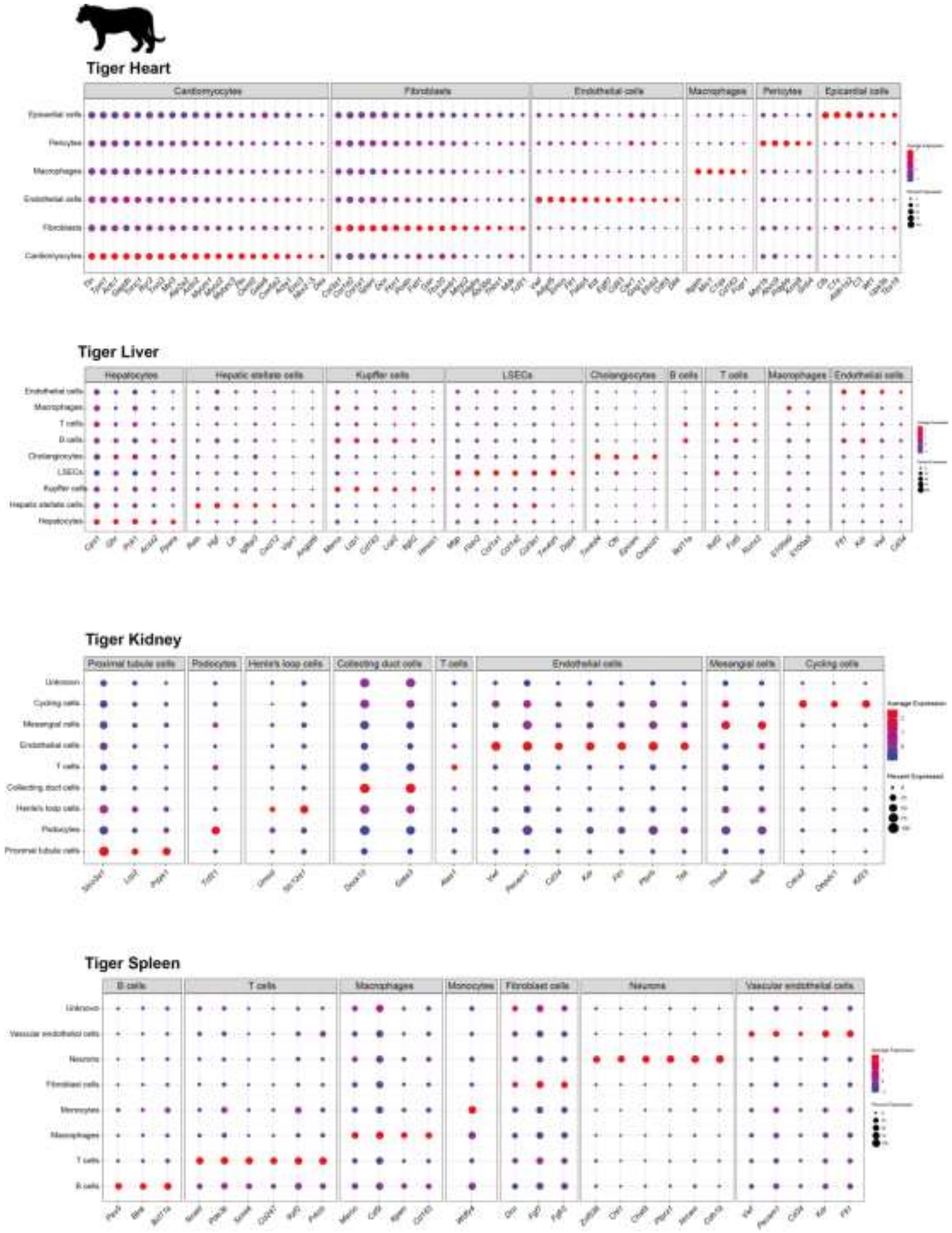

Supplementary Fig. 2. Dot plots showing expression patterns of cell type marker genes in different tissues of tiger.

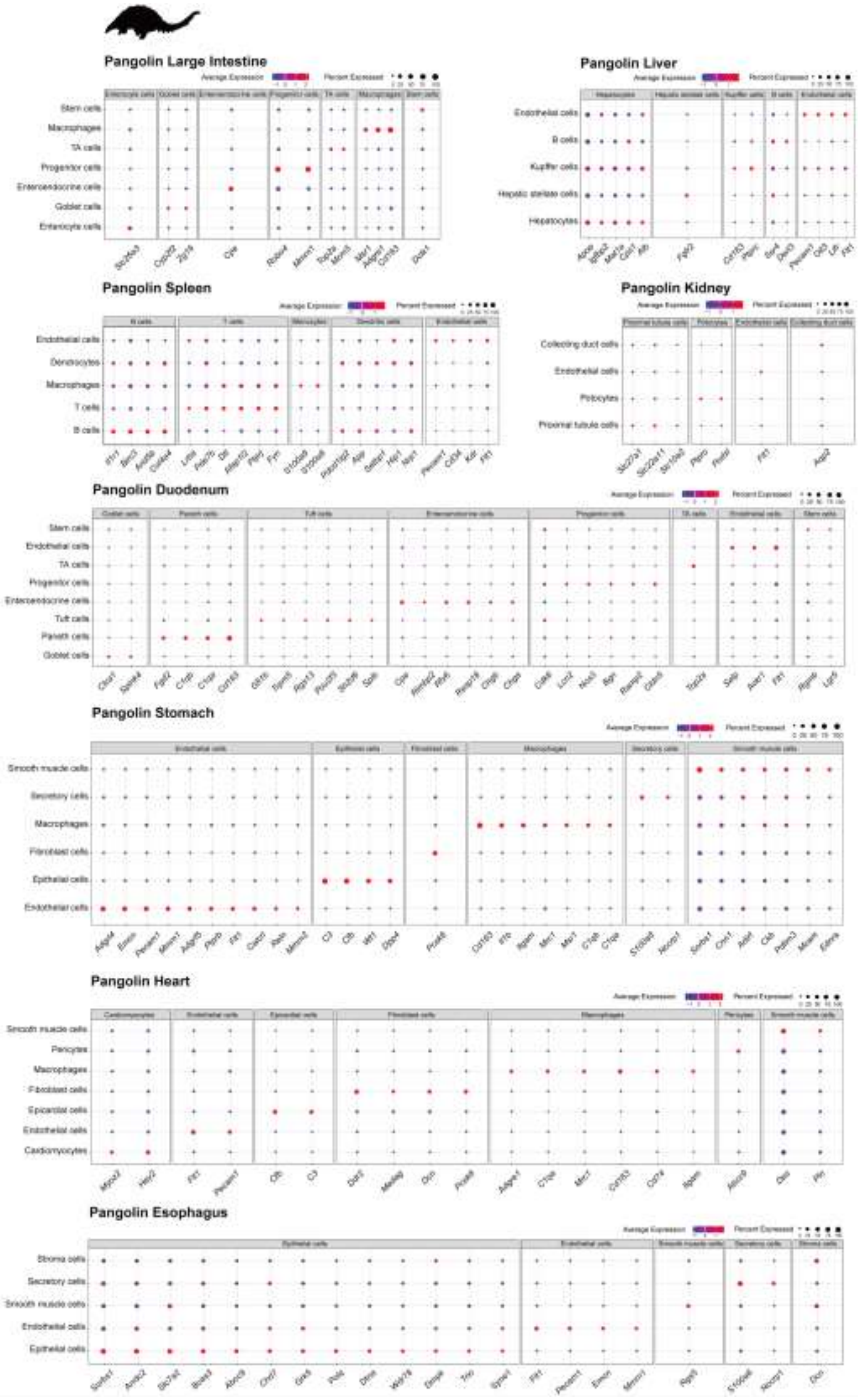

Supplementary Fig. 3. Dot plots showing expression patterns of cell type marker genes in different tissues of pangolin.

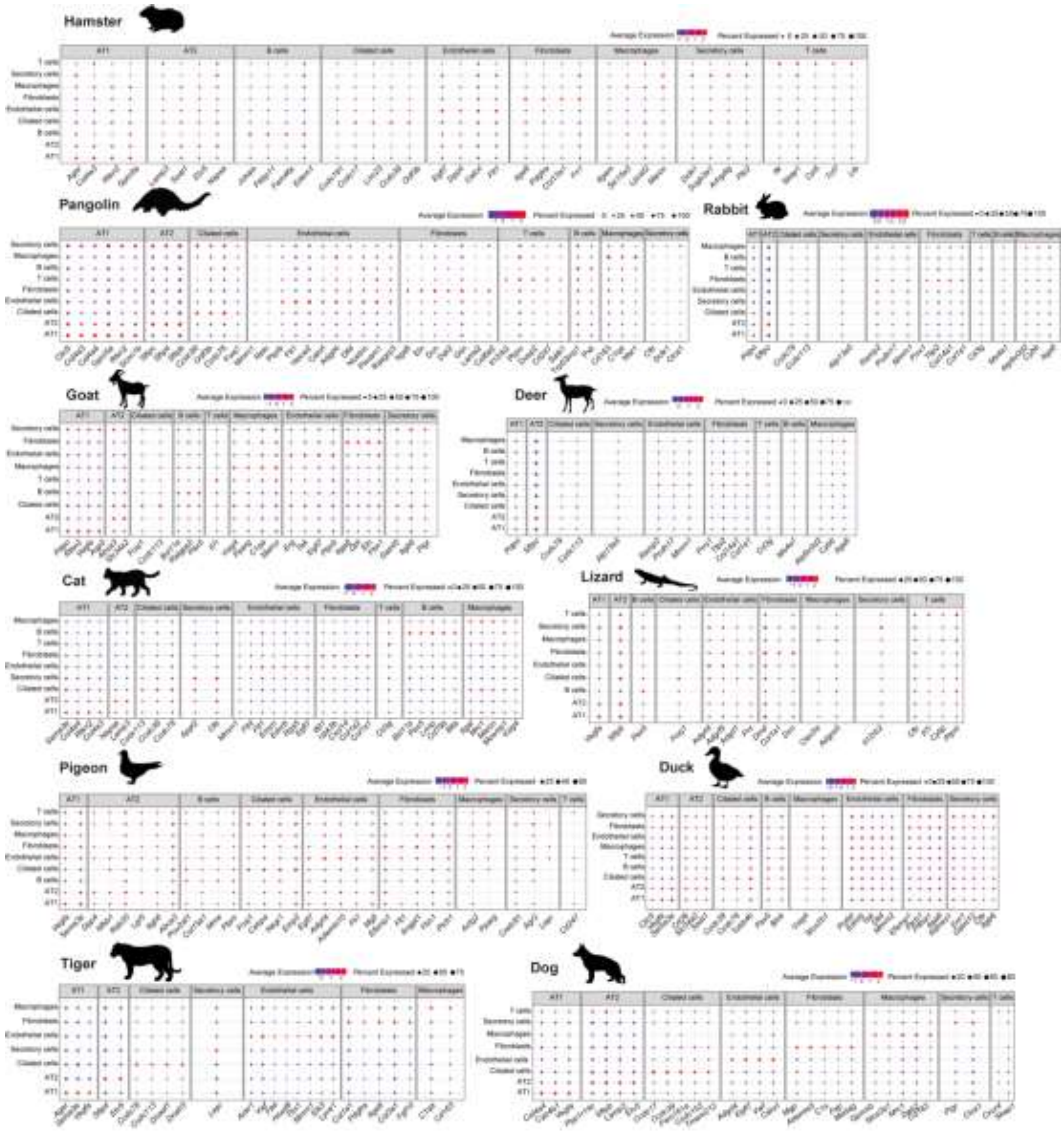

Supplementary Fig. 4. Cell type annotation of comparative lung atlas. Dot plots showing expression patterns of cell type marker genes in lung cells of different species.

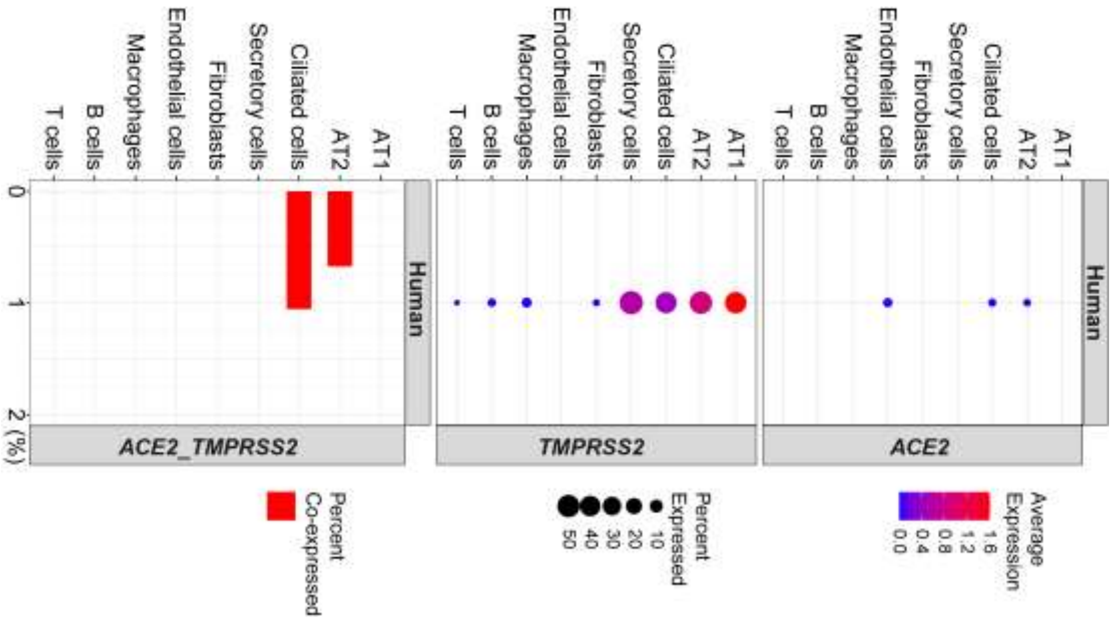

Supplementary Fig. 5. Ratio of cells expressing *ACE2*, *TMPRSS2* or both.

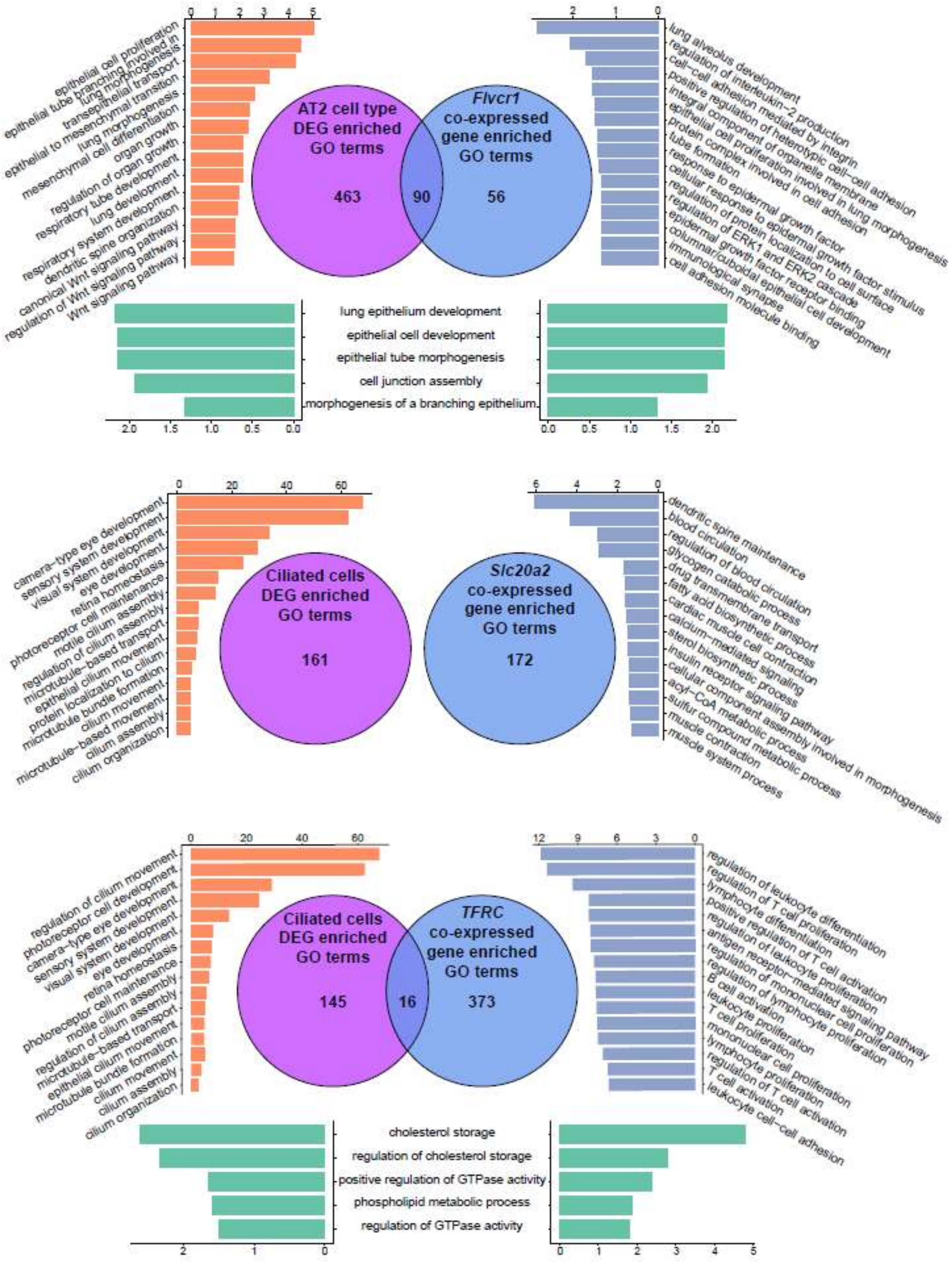

Supplementary Fig. 6. Shared and specific GO terms between viral receptor co-expressing genes and cell type DEGs. P values were calculated using hypergeometric test. Multiple comparisons adjustment was performed using Benjamini & Hochberg method.

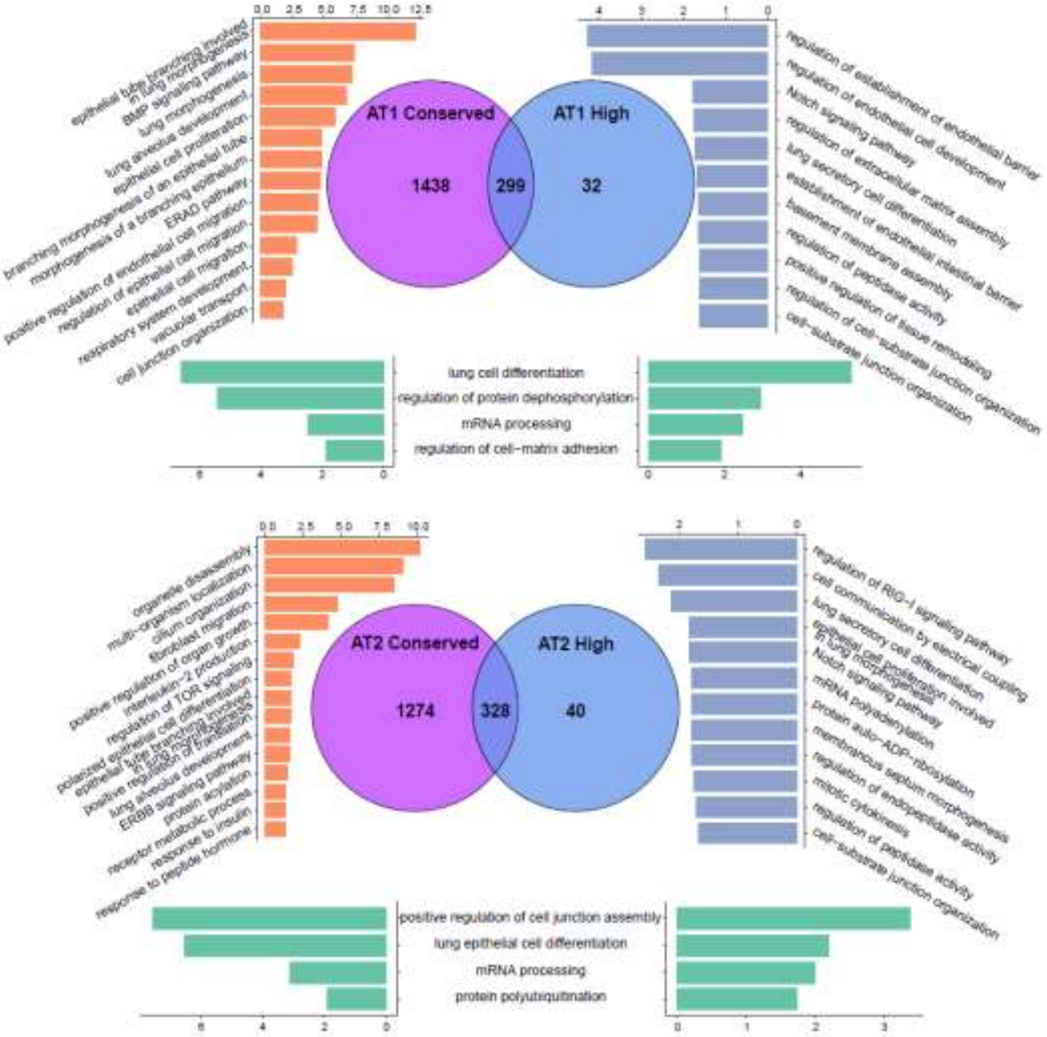

Supplementary Fig. 7. Shared and specific GO terms between AT1/2 conserved genes and AT1/2 highly expressed genes. P values were calculated using hypergeometric test. Multiple comparisons adjustment was performed using Benjamini & Hochberg method.
