# Supplementary material for: Single cell atlas for 11 non-model mammals, reptiles and birds
Source: Nat Commun. 2021 Dec 6;12:7083. doi: 10.1038/s41467-021-27162-2 (PMC8648889; doi:10.1038/s41467-021-27162-2)
Supplement: Supplementary file 2 — Reporting Summary [file 41467_2021_27162_MOESM2_ESM.pdf]

Corresponding author(s): Yan Hua, Yahong Liu, Huan Liu, Xun Xu

Last updated by author(s): Jul 26, 2021

Reporting Summary

Nature Portfolio wishes to improve the reproducibility of the work that we publish. This form provides structure for consistency and transparency in reporting. For further information on Nature Portfolio policies, see our Editorial Policies and the Editorial Policy Checklist.

Statistics

For all statistical analyses, confirm that the following items are present in the figure legend, table legend, main text, or Methods section.

n/a	Confirmed	
☐	☒	The exact sample size (n) for each experimental group/condition, given as a discrete number and unit of measurement
☐	☒	A statement on whether measurements were taken from distinct samples or whether the same sample was measured repeatedly
☐	☒	The statistical test(s) used AND whether they are one- or two-sided<br>*Only common tests should be described solely by name; describe more complex techniques in the Methods section.*
☐	☒	A description of all covariates tested
☐	☒	A description of any assumptions or corrections, such as tests of normality and adjustment for multiple comparisons
☒	☐	A full description of the statistical parameters including central tendency (e.g. means) or other basic estimates (e.g. regression coefficient) AND variation (e.g. standard deviation) or associated estimates of uncertainty (e.g. confidence intervals)
☐	☒	For null hypothesis testing, the test statistic (e.g. F, t, r) with confidence intervals, effect sizes, degrees of freedom and P value noted<br>*Give P values as exact values whenever suitable.*
☒	☐	For Bayesian analysis, information on the choice of priors and Markov chain Monte Carlo settings
☐	☒	For hierarchical and complex designs, identification of the appropriate level for tests and full reporting of outcomes
☐	☒	Estimates of effect sizes (e.g. Cohen's d, Pearson's r), indicating how they were calculated

Our web collection on statistics for biologists contains articles on many of the points above.

Software and code

Policy information about availability of computer code

Data collection	n/a
Data analysis	Custom scripts for data analysis in this study were present in https://github.com/DongshengChen-TY / Atlas (DOI: 10.5281/zenodo.5139392)

For manuscripts utilizing custom algorithms or software that are central to the research but not yet described in published literature, software must be made available to editors and reviewers. We strongly encourage code deposition in a community repository (e.g. GitHub). See the Nature Portfolio guidelines for submitting code & software for further information.

Data

Policy information about availability of data

All manuscripts must include a data availability statement. This statement should provide the following information, where applicable:

- Accession codes, unique identifiers, or web links for publicly available datasets
- A description of any restrictions on data availability
- For clinical datasets or third party data, please ensure that the statement adheres to our policy

The raw data and processed data generated in this study have been deposited in the NCBI database under accession code PRJNA747757 [https://www.ncbi.nlm.nih.gov/bioproject/PRJNA747757] and GEO database under accession code GSE183300 [https://www.ncbi.nlm.nih.gov/geo/query/acc.cgi?acc=GSE183300]. Alternatively, raw transcriptome sequencing data and processed data were deposited at the CNSA (CNGB Nucleotide Sequence Archive) under accession number CNP0001882 [https://db.cngb.org/search/project/CNP0001882] and CNP0001889 [https://db.cngb.org/search/project/CNP0001889].

Field-specific reporting

Please select the one below that is the best fit for your research. If you are not sure, read the appropriate sections before making your selection.

- [x] Life sciences
- [ ] Behavioural & social sciences
- [ ] Ecological, evolutionary & environmental sciences

For a reference copy of the document with all sections, see nature.com/documents/nr-reporting-summary-flat.pdf

Life sciences study design

All studies must disclose on these points even when the disclosure is negative.

Sample size	Tissue samples were obtained from 11 animals, including: 1) four pet species: cat (Felis catus), dog (Canis lupus familiaris), hamster (Mesocricetus auratus), and lizard (Anolis carolinensis); 2) two livestock species: goat (Capra aegagrus hircus) and rabbit (Oryctolagus cuniculus domesticus); 3) two poultry species: duck (Anas platyrhynchos domesticus) and pigeon (Columba livia domestica); and 4) three wild animal species: tiger (Panthera tigris altaica), pangolin (Manis javanica), and deer (Cervus nippon)
Data exclusions	No data were excluded from the analyses
Replication	Experimental replication was not attempted
Randomization	Randomization was not performed for this study
Blinding	Data collection and analysis were not performed blind to the conditions of the experiments

Reporting for specific materials, systems and methods

We require information from authors about some types of materials, experimental systems and methods used in many studies. Here, indicate whether each material, system or method listed is relevant to your study. If you are not sure if a list item applies to your research, read the appropriate section before selecting a response.

Materials & experimental systems

n/a	Involved in the study	
x		Antibodies
x		Eukaryotic cell lines
x		Palaeontology and archaeology
	x	Animals and other organisms
x		Human research participants
x		Clinical data
x		Dual use research of concern

Methods

n/a	Involved in the study	
x		ChIP-seq
x		Flow cytometry
x		MRI-based neuroimaging

Animals and other organisms

Policy information about studies involving animals; ARRIVE guidelines recommended for reporting animal research

Laboratory animals	n/a
Wild animals	cat (Felis catus), dog (Canis lupus familiaris), hamster (Mesocricetus auratus), lizard (Anolis carolinensis), goat (Capra aegagrus hircus), rabbit<br>(Oryctolagus cuniculus domesticus), duck (Anas platyrhynchos domesticus) , pigeon (Columba livia domestica), tiger (Panthera tigris altaica), pangolin (Manis javanica), deer (Cervus nippon)
Field-collected samples	n/a
Ethics oversight	All experimental procedures and sample collection protocols were performed with the approval of the Institutional Review Board on Ethics Committee of BGI (NO. BGI-IRB A20008).

Note that full information on the approval of the study protocol must also be provided in the manuscript.
