# Supplementary material for: Single cell atlas for 11 non-model mammals, reptiles and birds
Source: Nat Commun. 2021 Dec 6;12:7083. doi: 10.1038/s41467-021-27162-2 (PMC8648889; doi:10.1038/s41467-021-27162-2)
Supplement: Supplementary file 3 — Description of Additional Supplementary Files [file 41467_2021_27162_MOESM3_ESM.pdf]

# Description of Additional Supplementary Files

**File Name: Supplementary Data 1**

**Description: Quality control of single-nucleus sequencing libraries. a**, Quality control information of snRNA-seq data of 11 species. **b**, Reference genome version. **c,** Cluster-specific marker genes of cat tissues. **d,** Cluster-specific marker genes of tiger tissues**. e,** Cluster-specific marker genes of pangolin tissues**. f,** Cluster-specific marker genes of lung in 11 species. P values were calculated using the Wilcoxon rank sum test. Multiple comparisons adjustment was performed using the Bonferroni correction.

**File Name: Supplementary Data 2**

**Description: Detailed expression percentage of cells co-expressing *ACE2* and *TMPRSS2* within each cell type in cat (a), tiger (b), and pangolin (c).**

**File Name: Supplementary Data 3**

**Description: Detailed expression percentage of *ACE2*, *TMPRSS2*, and their co-expression in comparative lung atlas. a,** Expression ratio of *ACE2* and *TMPRSS2* within each cell type of lungs in 11 species **b,** Co-expression ratio of *ACE2* and *TMPRSS2* within each cell type of lungs in 11 species.

**File Name: Supplementary Data 4**

**Description: List of selected feline viruses and corresponding receptors. a,** List of feline viruses infecting cats and corresponding receptors. **b,** Expression ratio of feline virus receptors within each cell type of tissues in cat. **c,** GO terms enriched from top 100 correlated genes for feline virus receptors. P values were calculated using hypergeometric test. Multiple comparisons adjustment was performed using Benjamini & Hochberg method.

**File Name: Supplementary Data 5**

**Description: Ligand receptor-mediated interaction network in lungs of different species. a,** cat. **b,** tiger. **c,** pangolin. **d,** dog. **e,** duck. **f,** deer. **g,** hamster. **h,** lizard, and **i**, goat. P values were calculated using Wilcoxon rank sum test. Multiple comparisons adjustment was performed using the Bonferroni correction.

**File Name: Supplementary Data 6**

**Description: Conservation of pulmonary cellular interactions. a-b,** Cellular interactions detected in all nine species. **c,** 642 pairs of pan-conserved cellular interactions. **d,** 147 pairs of cellular interactions conserved in a mammalian-specific manner.

**File Name: Supplementary Data 7-15**

**Description: List of TF-target pairs.** List of all TF-target pairs in cat (Supplementary Data 7), tiger (Supplementary Data 8), pangolin (Supplementary Data 9), dog (Supplementary Data 10), hamster (Supplementary Data 11), deer (Supplementary Data 12), goat (Supplementary Data 13), lizard (Supplementary Data 14), and duck (Supplementary Data 15).

**File Name: Supplementary Data 16**

**Description: Conserved TF-target pairs (>=2 species) in nine cell types.**

**File Name: Supplementary Data 17**

**Description: Highly conserved (>=4 species) TF-target pairs in all cell types.**

**File Name: Supplementary Data 18**

**Description: GO enrichment of predicted target genes of top 30 TFs in nine cell types.** P values were calculated using hypergeometric test. Multiple comparisons adjustment was performed using Benjamini & Hochberg method.

**File Name: Supplementary Data 19**

**Description: a,** Expression percentile matrix of 5 442 candidate genes. **b,** Gene clusters of AT1 cells. **c**, Gene clusters of AT2 cells.

**File Name: Supplementary Data 20**

**Description: a,** GO term enrichment in AT1 cell conserved core gene expression program. **b,** GO term enrichment in AT2 cell conserved core gene expression program. P values were calculated using hypergeometric test. Multiple comparisons adjustment was performed using Benjamini & Hochberg method.

**File Name: Supplementary Data 21**

**Description: Statistical test of *ACE2* & *TMPRSS2* co-expression ratio between cat ciliated cells and those of other species.** P values were calculated using the Wilcoxon rank sum test.

**File Name: Supplementary Data 22**

**Description: Cell type enrichment of *ACE2* & *TMPRSS2* co-expression.** P values were calculated using the Wilcoxon rank sum test.
